# The Glymphatic System (En)during Inflammation

**DOI:** 10.3390/ijms22147491

**Published:** 2021-07-13

**Authors:** Frida Lind-Holm Mogensen, Christine Delle, Maiken Nedergaard

**Affiliations:** 1 Center for Translational Neuromedicine, Faculty of Health and Medical Sciences, University of Copenhagen, 2200 Copenhagen, Denmark; Frida.lind-holm@lih.lu (F.L.-H.M.); christine.delle@sund.ku.dk (C.D.); 2 Center for Translational Neuromedicine, University of Rochester Medical Center, Rochester, NY 14642, USA

**Keywords:** glymphatic system, astrocytes, glia limitans, AQP4, ocular glymphatic system, inflammation, immune privilege, CSF, ISF, immune surveillance

## Abstract

The glymphatic system is a fluid-transport system that accesses all regions of the brain. It facilitates the exchange of cerebrospinal fluid and interstitial fluid and clears waste from the metabolically active brain. Astrocytic endfeet and their dense expression of the aquaporin-4 water channels promote fluid exchange between the perivascular spaces and the neuropil. Cerebrospinal and interstitial fluids are together transported back to the vascular compartment by meningeal and cervical lymphatic vessels. Multiple lines of work show that neurological diseases in general impair glymphatic fluid transport. Insofar as the glymphatic system plays a pseudo-lymphatic role in the central nervous system, it is poised to play a role in neuroinflammation. In this review, we discuss how the association of the glymphatic system with the meningeal lymphatic vessel calls for a renewal of established concepts on the CNS as an immune-privileged site. We also discuss potential approaches to target the glymphatic system to combat neuroinflammation.

## 1. Introduction

The blood–brain barrier (BBB) is a critical component of the central nervous system (CNS) that dampens environmental perturbations in interstitial ionic and chemical concentrations, and thereby supports the stability of synaptic transmission. Long-distance neural networks require a protected environment with limited fluctuations of the concentrations of interstitial ions, neurotransmitters, essential metabolic intermediates, and toxic xenobiotics. The BBB also restricts the entry to the brain of peripheral inflammatory mediators and pathogens that might negatively impact neuronal activity. In the eye, the blood–retina barrier (BRB) similarly protects the environment of the neuroretina. The CNS lacks a parenchymal lymphatic vessel system that supports fluid homeostasis and provides a path for immune surveillance in the peripheral organs. The lack of lymphatic vessels in the retina and brain presented a conceptual challenge in understanding how fluid homeostasis and the export of waste products are obtained within the confines of the CNS [1]. With the discovery of the glymphatic system initially in brains of rodents [2,3,4,5], and later in human brain by magnetic resonance imaging (MRI) studies [6,7,8,9,10], a glial-associated functional homologue of the lymphatic system was established. More recently, the existence of an ocular glymphatic clearance system was demonstrated [11]. Astrocytes play a key role in the glymphatic system. Astrocytes create with their vascular endfeet the perivascular spaces that surround the cerebral vasculature. The perivascular spaces are utilized as “highways” for fast transport of cerebrospinal fluid (CSF) into deep brain regions. 

The heterogenic nature and function of astrocytes in neurological diseases has been studied excessively at molecular and cellular levels. Many excellent reviews exist on this topic [12,13,14]. The existence of the glymphatic system provides, however, a novel perspective on the role of astrocytes in pathological processes: Astrocytes are not only participants on a microscopic level in disease processes, but contribute macroscopically to pathology through glymphatic impairment. This review focuses on the pivotal function of astrocytes in fluid transport and as global players for maintaining CNS homeostasis in the setting of either inflammatory processes in CNS or in systemic inflammation.

Only a few years after the initial description of the brain glymphatic system, the research groups of Kipnis and Alitalo independently discovered a lymphatic vessel network in the cerebral meninges. The meningeal lymphatic system serves traditional functions such as immune-cell trafficking and clearance of macromolecules from the brain [15,16]. The glymphatic network is a fluid-clearance system that drains into the dural lymphatic network ensheathing the brain, and onward to lymph vessels that track the cranial nerves and large vessels [15,16,17].

We have come to understand that the neuroimmune interface of the glymphatic/lymphatic system allows immune surveillance of the CNS [18]. Yet, there is a pressing need to investigate the exact roles of the glymphatic and meningeal lymphatic system in the contexts of acute and chronic neuroinflammation. We herein propose possible functions of and interactions between the two systems. Further, we discuss how recent discoveries call for a revision of the classic concept of CNS immune privilege and how peripheral inflammation may propagate to induce neuroinflammation.

### 1.1. The Brain Glymphatic System

**Figure 1:**
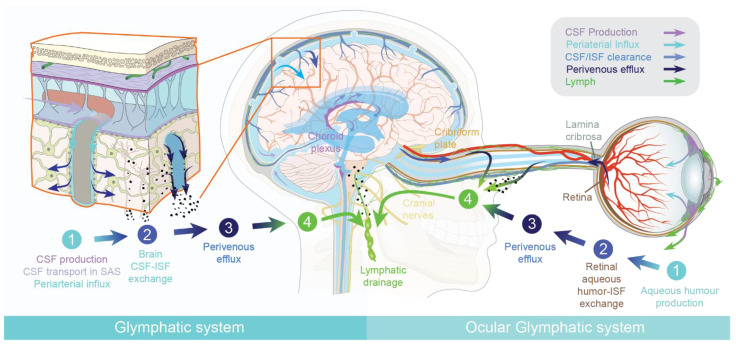
The glymphatic systems in the brain and the eye export fluid and solutes from metabolically active neural tissue. The organization of brain and ocular fluid flow can be divided into four distinct segments that share similarities, but differ in specific respects. (1) The first segment of the brain glymphatic system includes CSF production (purple arrows) and circulation in the subarachnoid space (SAS, light purple arrow), followed by periarterial influx of CSF into the brain tissue (light blue arrows). Two influx paths exist in the eye: the first path is the ciliary body, which produces the aqueous humor fluid. Most of the aqueous humor fluid leaves the eye via the anterior chamber. However, aqueous humor also moves posteriorly, passing the vitreal body before entering the neuroretina to support removal of waste products from that metabolically active tissue. The second inflow path is limited to periarterial influx of CSF along the posterior segment of the optic nerve. CSF does not pass into the eye, but drains into lymphatic vessels located in the dural sheet surrounding the optic nerve prior to the lamina cribrosa barrier [11,60]. (2) The second segment of the brain glymphatics is CSF–ISF exchange supported by AQP4 channels in the vascular endfeet plastered along the arterioles (blue arrows). In the eye, the aqueous humor mixes with interstitial fluid and is transported along axons across the lamina cribrosa barrier. From here the fluid leaves the axons and moves towards the perivenous space in a path supported by astrocytes. Astrocytic AQP4 water channels facilitate this segment as deletion of AQP4 suppresses ocular glymphatic activity [11]. (3) The third segment of the glymphatic system, which is common to the brain and eye, consists of perivenous efflux of interstitial fluid (ISF, dark blue arrows), which drains to the dural lymphatic vessels surrounding the brain and the optic nerve (green). (4) Ultimately, the drainage of perivenous waste from the eye ends up in the cervical lymph nodes (green), which constitute the fourth segment of the fluid-transport system. Fluids from both the brain and the eye thus drain via the cervical lymphatic vessels, which empty into the venous system at the level of the subclavian veins.

In peripheral tissues, leaky capillaries in peripheral tissues allow a constant influx of a plasma ultrafiltrate. Inflowing fluid support distribution and energy metabolites, as well as signaling molecules. Within the tissue, the plasma ultrafiltrate mixes with interstitial fluid (ISF), while excess fluid is transported away by the lymphatic system [19]. Thus, lymphatic vessels maintain fluid homeostasis and are especially dense in areas with a high metabolic demand [1,20]. In the brain and the eye, influx of a plasma ultrafiltrate is limited due to the presence of the blood–brain barrier (BBB) and blood–retinal barrier. Instead, both tissues produce their own fluid, cerebrospinal fluid (CSF) and aqueous humor, respectively. Both the brain and the eye were long believed to be devoid of a lymphatic vascular system. The brain is among the most metabolically active organs, and it was therefore difficult to understand how it could maintain homeostasis in the absence of parenchymal lymphatic vessels [21]. In 2012, there was the first report of a fluid-transport system in the brain, which was designated the glial-associated lymphatic system, or the “glymphatic system” [3]. The glymphatic system consists of three functional compartments, each facilitating movement of cerebrospinal fluid (CSF) and interstitial fluid (ISF) in the brain (Figure 1). **The first compartment, the glymphatic influx**, occurs in the subarachnoid space, where CSF enters by bulk flow into the periarterial spaces surrounding the arteries that penetrate deep into the brain parenchyma [3]. **The second compartment**, **the exchange of CSF and ISF**, occurs in the interstitial space of the brain parenchyma. Here, the movement of CSF into the parenchyma is facilitated by the aquaporin-4 (AQP4) water channels abundantly expressed on astrocytic endfeet lining the peri-arterial spaces [3,22]. These endfeet ensheath the cerebral vasculature and express AQP4 in a polarized manner towards the basal lamina of the perivascular spaces. This arrangement facilitates the rapid movement of water between the perivascular space and the glial syncytium, and thereby supports perivascular fluid and solute movement along the glymphatic system [23]. Aquaporins increase the rate of water influx into cells by 10–100-fold in excess of influx by other mechanisms and ion channels [24]. In the brain, aquaporin 1 is expressed by the choroid plexus ependymal cells, but AQP4 is the principal water channel in the parenchyma and a key component of glymphatic clearance. AQP4 is astrocyte-specific in the human and rodent brain [24,25,26,27,28].

The glymphatic system is impaired in AQP4-deficient mice, suggesting that AQP4 plays a key role in facilitating the perivascular influx of CSF [3,22]. Nevertheless, the exact mechanism by which AQP4 supports brain fluid transport remains unknown. The exchange between CSF and ISF happens in the interstitial space of the brain parenchyma, where dispersion of ISF drives waste products towards the perivascular spaces on the venous side for efflux from the CNS. Fluid transport in the perivascular space occurs by convective flow, whereas transport in the interstitial is a mixture of diffusion and convection [9,29,30]. **The third compartment, the glymphatic efflux**, consists of drainage of interstitial fluid into the perivenous space, from which neurotoxic and metabolic wastes from the ISF either re-enter the CSF or are transported directly out along meningeal and cervical lymphatic vessels, and along cranial and spinal nerves (Figure 1).

The known driving forces of glymphatic flow are arterial pressure waves generated by cardiac pulsatility, respiration, and slow vasomotion [31,32,33,34]. Glymphatic flow is primarily activated during sleep [35] and by certain anesthetics that promote slow-wave activity (1–4 Hz, so-called Delta waves) [33]. In addition, the efficiency of the glymphatic system is affected by sleeping posture, being most effective in the lateral and supine head position [4,33,36]. Aging and chronic diseases suppress glymphatic function [37,38,39]. In neurodegenerative diseases, such as Alzheimer’s disease (AD), and to a lesser extent with normal aging, the glymphatic system is impaired, due at least in part to the dysregulated expression of AQP4 on the astrocytic endfeet [37,40,41]. The effect of chronic diseases on glymphatic flow has been discussed in several recent reviews [17,41,42,43,44,45,46].

Located downstream of the glymphatic system, the meningeal lymphatic system can be viewed as the fourth compartment of brain fluid transport [15,16]. This system of lymph vessels in the meninges drains waste, signaling molecules, and other solutes from the CNS that are transported by the glymphatic system. The processes that govern solute exchange between interstitial fluid, cerebrospinal fluid, and the meningeal lymphatic compartments are a matter of intensive current research. In particular, it remains to be established how exactly the glymphatic system interconnects with the meningeal lymphatic system and the (peripheral) immune system.

### 1.2. The Ocular Glymphatic System

The retina is an extension of the brain and, as such, largely lacks lymphatic vessels [47,48]. Similarly to the brain, the eye produces its own fluid, known as the aqueous humor. Aqueous humor drains out of the eye via the anterior chamber and Schlemm’s canal, although some fluid can also leave by uveoscleral drainage [49,50]. Yet, the neuroretina in the back of the eye is metabolically the most active part of the eye, and is thus in need of a fluid-transport mechanism to remove neurotoxic waste products. The initial description of the brain´s glymphatic pathway [3] prompted several groups to suggest that the eye similarly houses a fluid-transport system specifically designed to clear the retina of metabolic waste products [51,52]. In fact, older studies had described fluid movements in the posterior part of the eye of multiple species, but had not related this to waste clearance [53,54,55,56,57,58,59]. The existence of an ocular glymphatic clearance system has now been demonstrated in rodents [11], underlining the general requirement of fluid transport in metabolically active neural tissues. Similarly to the brain glymphatic system, the ocular glymphatic clearance system clears fluid and solutes and is divided into four functional segments. the aqueous humor is produced by the ciliary body (first segment) and enters the neuroretina after passing through the vitreous body (second segment). The constant influx of aqueous humor into the metabolically active neuroretina is the most important segment of the ocular glymphatic system. Here, the aqueous humor mixes with interstitial retinal fluid. The excess fluid is transported along the axons of retinal ganglion cells across the lamina cribrosa barrier, from which the fluid accumulates in the perivenous spaces supported by AQP4 channels (third segment). This process is at least in part driven by the ocular–cranial pressure difference, which is normally positive due to the physiologically higher intraocular pressure relative to the intracranial pressure. Light-induced pupil constriction accelerates the movement of intraocular tracers into the optic nerve, possibly supported by the minor pressure pulses arising from smooth muscle constriction [11]. Intraocularly administered tracers (e.g., amyloid-β) exit the eye along the axons of retinal ganglion cells, and then enter perivenous spaces before draining through lymphatic vessels (fourth segment) into the cervical lymph nodes [11]. It is important to note that fluid transport in the posterior segment of the optic nerve close to the brain is supported by CSF influx along the central retinal artery [11,60]. However, the periarterial influx of CSF does not reach the eye, but is drained along the dural lymphatic vessels, similarly to aqueous humor efflux from the eye [11]. Thus, CSF tracers do not enter the eye, even in glaucoma models, and (like the aqueous humor) leave the optic nerve via dural lymphatic vessels. In anatomic terms, the brain and optic nerves are ensheathed in the same meningeal–dural membrane, and thus share the same CSF pool. The main difference between the brain and the ocular glymphatic system is the source of fluid entering the neuropil; i.e., CSF in the brain and aqueous humor fluid in the retina. In other respects, the polarized fluid transport is organized in a similar manner.

## 2. How Does Neuroinflammation Affect the Glymphatic System?

Among the various pathological events that can lead to neuroinflammation are traumatic brain injury (TBI) [61], meningitis triggered by either viral or bacterial invasion [62], autoimmune diseases such as multiple sclerosis [63], and neurodegeneration [64,65,66,67,68,69]. An impairment of CSF flow has been described for most chronic neurological diseases of the brain, including TBI, stroke, Alzheimer′s disease, and Parkinson’s disease [2,41,42,70,71,72], whereas acute stroke is linked to a transient acceleration of CSF inflow [73]. A plausible general scenario in the setting of neuroinflammation is that glymphatic impairment aggravates inflammation by suppressing cytokine clearance from the brain [74].

The reactive astrogliosis resulting from an inflammatory insult [69,75] likely reduces brain clearance, as astrocytes are immunocompetent cells that produce cytokines and other inflammatory mediators [69,76,77,78,79] and thus contribute to neuroinflammation. In parallel, reactive changes of microglial cells and astrocyte morphology would plausibly lead to an additional slowing of glymphatic flow. Interestingly, acute inflammation is linked to CSF hypersecretion, as shown in a recent study addressing how the choroid plexus reacts to local and peripheral inflammation [80]. It seems self-evident that any changes in CSF production could influence the brain’s drainage systems, but exactly how this might be obtained remains to be elucidated. Of note, Kähle and colleagues reported that the Toll-like receptor 4 (TLR-4) signaling pathway is crucial in activating the Na+, K+, Cl− cotransporter (NKCC1), resulting in hypersecretion of CSF from the choroid plexus epithelium in a model of post-hemorrhagic hydrocephalus. The hypersecretion of CSF peaked at 24 h, but was still elevated 48 h after the injury [80]. It is possible that transient CSF hypersecretion leads to an initially increased drainage of fluid, waste, and inflammatory mediators to the periphery, which is a driver for activating and attracting peripheral immune cells to the meningeal lymphatic vessels.

CSF hypersecretion in response to local injury will likely enhance clearance of antigens (cell debris, viruses, or bacteria) and thereby support a rapid immune response. At the same time, acute inflammation is also linked to an opening of the BBB and subsequent infiltration of immune cells into the CNS parenchyma [63]. Classically defined neuroinflammatory diseases, including multiple sclerosis or bacterial meningitis, are similarly characterized by a breakdown of the BBB [81], leaving the brain exposed to peripheral factors.

AQP4 knock-out mice demonstrate reduced glymphatic fluid transport, possibly explaining why experimental models of meningitis and multiple sclerosis; i.e., the experimental autoimmune encephalomyelitis model, exhibit less-severe symptoms [82,83]. It is possible that reduced glymphatic efflux of antigens will reduce the severity of the immune response, resulting in less tissue swelling and therefore milder neurological symptoms. This observation seems to contradict the finding that AQP4 expression increases during inflammatory conditions, including treatment with bacterial lipopolysaccharides (LPS) [84]. Here it is important to recall that an increase in AQP4 expression in the setting of inflammation or injury does not support glymphatic fluid transport. The additional AQP4 channels are not inserted in the vascular endfeet of astrocytes, but rather in the cell body and perisynaptic processes. The subsequent loss of the vascular polarization of AQP4 correlates with a *decrease* in glymphatic flow [37]. Similarly, a loss of AQP4 vascular polarization has been noted in aging and several other conditions marked by chronic neuroinflammation [37,70,85]. In fact, genome-wide association studies, longitudinal patients’ studies, as well as genetically modified animal models, indicate that immune activation and general neuroinflammation occur early in the course of neurodegenerative diseases [42,64,67,86,87,88,89,90]. We propose that long-lasting reduction in glymphatic activity accelerates disease progression in a range of contexts.

**Figure 2:**
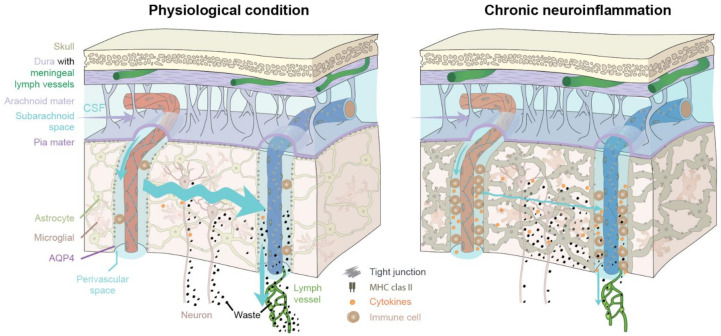
Neuroinflammation impairs glymphatic function and exacerbates the inflammatory response.

In addition to altered AQP4 polarization, infiltrating immune cells are known to accumulate in the perivascular spaces during inflammation, and may then physically block perivascular flow and influx of CSF [18,91,92]. It is possible that the impairment of the glymphatic flow and resultant accumulation of cytokines [93] and metabolic wastes create a vicious cycle to perpetuate neuroinflammation (see Figure 2). In the context of meningeal lymphatic vessels, recent work showed that ablation of drainage through the meningeal lymphatic vessels in a mouse model of Alzheimer’s disease exacerbated amyloid-β deposition, neurovascular dysfunction, microgliosis, and behavioral deficits [94]. Interestingly, microglia changed towards a more inflammatory phenotype when meningeal lymphatic vessels were ablated [94]. These findings underscore our hypothesis that an impairment of the brain’s drainage system accelerates the neuroinflammatory response, probably due to the accumulation or entrapment of waste and pro-inflammatory cytokines within the brain. Another study showed that ligation of the deep cervical lymph nodes in AQP4−/− mice aggravated their brain pathology, manifesting in microglial activation and hippocampal neuronal apoptosis, while also leading to impaired exploratory and cognitive abilities compared to wild-type mice [95].

### 2.1. How Does Peripheral Systemic Inflammation Lead to Neuroinflammation?

Peripheral inflammation refers to any activation of the innate or adaptive immune system outside of the CNS [81]. An initial peripheral infection can perturb CNS function, with responses ranging from small perturbations in body temperature, to severe fatigue and loss of consciousness, as can occur in systemic infections. How the brain and the periphery communicate during systemic inflammation is poorly understood. Short-term acute inflammation does not normally affect the homeostasis of the brain, thanks to the defense afforded by an intact BBB [96,97]. However, severe peripheral inflammation can often involve the CNS and trigger neuroinflammation [65]. There are at least five ways whereby a peripheral inflammation can come to involve the CNS [98,99]. First, circulating cytokines can activate capillary endothelial cells [100,101,102], which then secrete cytokines into the perivascular spaces, followed by their transport to all parts of the brain via the operation of the glymphatic system. Second, it may be that certain cytokines are transported across the BBB, although this process is likely to vary across brain regions and physiological conditions, and between different cytokines [96,99,103,104]. In addition, the expression of putative BBB transporters may change during inflammation [81,96], although it is certainly the case that cytokines can cross into the CNS when the BBB is impaired or compromised [81,96]. Third, cytokines and chemokines are known to communicate with and activate the circumventricular organs [105], which allow entry of low-molecular-weight molecules from circulation due to their low expression of gap junctions compared to the BBB [106,107]. The fourth known entry point is through activation of peripheral nerves via cytokines [108,109]. Finally, the choroid plexus, with its fenestrated capillaries, may also be an entry point for foreign pathogens and immune cells in circulation [98,110,111]. The choroid plexus is essential for brain fluid homeostasis [111], and recent studies show that it may communicate with glial cells resident in the adjacent brain parenchyma via choroid plexus-derived extracellular vesicles [112]. Additionally, it is apparent that peripheral inflammation affects behavior, sleep, memory, and cognition. There is an abundant literature showing that peripheral inflammation, perhaps by secondary involvement of CNS, can contribute to neuronal damage and increase the risk of neurodegenerative processes [65,113,114].

A recent study elucidated how the glymphatic system reacts to a peripheral inflammatory challenge [115]. In that study, there was reduced perivascular CSF flow as soon as three hours after peripheral LPS treatment at a single dose of 1 mg/kg in mice [115]. Other factors known to influence the glymphatic function were measured; respiration, cortical blood flow, astrogliosis, and AQP4 polarization were surprisingly unchanged at three hours, despite the reduction in CSF flow. The study points towards another physiological response of the glymphatic system after endotoxin exposure, as only the heart rate and the microglia activation were elevated in these mice [115]. In support of a model of suppressed glymphatic function after LPS injection, Erickson et al. demonstrated reduced amyloid-β clearance 28 h after LPS exposure [116]. However, it is not fully elucidated how peripheral inflammation impacts the glymphatic system or the downstream clearance through meningeal lymphatic vessels. We believe that further investigation is warranted in this domain, as it could point towards important new therapeutic strategies for treating central inflammation.

In Figure 2 (left), the glymphatic system under physiological and healthy conditions is depicted. Only a very few perivascular immune cells are normally present in brain. The polarized expression of AQP4 towards the astrocytic endfeet facilitates the influx of CSF into the brain parenchyma. The interstitial space expands during sleep to enhance influx of CSF, which mixes with the interstitial fluid [35]. The excess fluid drains out along perivenous spaces and cranial nerves. This highly organized polarized pattern of fluid flow removes metabolic waste such as amyloid-β and cytokines from the brain parenchyma. The waste is ultimately drained into extracerebral cervical lymphatic vessels (green). In Figure 2 (right), chronic neuroinflammation impairs the flow of CSF into the brain parenchyma by several mechanisms, including accumulation of perivascular immune cells, and dysregulation of polarized AQP4 expression from the astrocytic endfeet towards the soma (loss of vascular polarity), resulting in reduced CSF influx. Both astrocytes and microglial cells undergo reactive morphological changes during an inflammatory response, contributing to reduced CSF/ISF exchange and fluid transport within the brain parenchyma, and resulting in edema formation. In turn, waste and cytokine accumulation will further drive inflammation, suppress glymphatic flow, and increase tissue swelling in a vicious cycle.

### 2.2. Neuromyelitis Optica Spectrum Disorders and AQP4

As described in Section 1.2, the brain and the optic nerves are ensheathed by the meninges and thus share the same CSF pool (see Figure 1). A bidirectional glymphatic transport along the optic nerves was demonstrated upon injection of tracer to the cisterna magna (CM) and the eye [11,60]. With AQP4 as a crucial water channel facilitating fluid fluxes in the CNS, neurological disorders affecting AQP4 physiology call for further research to investigate changes in CNS fluid dynamics and the impact of astrocytic dysfunction in neuroinflammation. Neuromyelitis optica (NMO) is a variety of rare autoimmune inflammatory disorders of the CNS mainly affecting the optic nerve and the spinal cord [117,118,119], collectively known as neuromyelitis optica spectrum disorders (NMOSD) [120]. Its main symptoms are optic neuritis, leading to vision loss and total blindness, and transverse myelitis, with poor or no potential for recovery. Optic neuritis and transverse myelitis are characterized by inflammation, immune cell infiltration, and swelling of the targeted tissues [117,118,119]. Furthermore, the intraocular pressure (IOP) appears to be elevated in patients with NMO, although their elevated IOP does not correlate with the extent of visual impairment [121]. In the past, NMO was regarded as a clinical variant of multiple sclerosis (MS) [122]. However, recent discoveries prove NMOSD to be a distinct disorder characterized by different features of MRI lesions in the brain and the spinal cord [123]. Brain regions with high AQP4 expression are especially affected in NMO, notably the hypothalamus [124]. Moreover, autoantibodies, mostly against AQP4 [118,125,126], are found in up to 80% of NMO individuals [127]. This distinguishes NMO as an individual auto-inflammatory disorder, unlike MS [118]. While a majority of NMO patients are reported to be serum-positive for AQP4 autoantibodies [128], myelin basic protein was recently described as potential biomarker of MS [129]. In MS, several subsets of autoantibodies target various neuronal or glial epitopes, but a clear diagnostic profile or their definite role in myelin loss is a matter of controversy to this day [130]. While NMOSD and MS are both inflammatory demyelinating disorders, in NMOSD, IgG autoantibodies mainly target astrocytes, without necessarily causing the loss of myelin or neuronal axons [120,131]. Notably, the pathology of MS is mainly characterized by attacks of the myelin sheaths, leading to demyelination and axonal loss. However, the NMOSD astrocytic attack causes prominent axonal swelling, which may possibly be an initiator of myelin loss [122]. The crucial role of astrocytes in MS was recently revised in a manner presenting astrocytes as a potential therapeutic target [132]. So far the role of the glymphatic system in NMO is unknown, but in a likely scenario, the autoantibodies that attack the glial AQP4 water channels, along with swelling of the optic nerve, will together suppress the ocular and brain glymphatic system [22]. NMO animal models would thus present a highly attractive tool to investigate the role of APQ4 and astrocytes on fluid dynamics in neuroinflammatory processes.

## 3. Revising the Immune Privilege of the CNS

Early studies that led to the conceptualization of the CNS as an “immunologically privileged site” involved implanting a tissue graft without provoking a subsequent immunological response and graft rejection [133,134]. Grafts and tumors transplanted in the brain or the anterior chamber of the eye survived without eliciting an immune response [133,135]. These pioneering studies presented the brain and the eye as tolerogenic organs that were not exposed to the immune system. Multiple characteristics of the specialized organization of CNS were thought to result in the typically slow or absent immune reactions occurring in the brain. The key hallmarks of this immune privilege are the physical barriers such as the BBB, the absence of professional antigen-presenting cells (APCs) in the brain parenchyma, and the expression of immunoregulatory proteins (Fas ligand, PD-1), together with the low expression of MHC class I and II molecules in the brain relative to expression in the periphery [136,137,138]. For decades it had been believed that “immune privilege” arose from the failure of antigens to leave the privileged sites and present to immunocompetent cells to evoke an immune response [136]. The brain, eyes, testis, and uterus (fetus) are still widely considered to be immunologically privileged organs of the human body [139,140]. As our knowledge of neuroimmune interactions expands, the mechanisms of immune privilege are often revisited and refined [15,138,141,142]. A key feature of immune privilege is that antigens can exit the organ to only a limited extent due to the lack of conventional lymph vessels [133]. The original studies by Medawar and Murphy were eventually called into question by reports that antigens inserted in the CNS can indeed leave the brain and subsequently induce immunological effects [143,144]. The notion of complete isolation of the CNS from the periphery and lack of immune responses was later disproven in studies showing eventual rejection of brain grafts [145]. Additional evidence is provided by findings that immune-deficient nude mice, or Rag1−/− mice that exhibit reduced numbers or lack mature T and B cells, can host xenografts of stem or cancer cells [146,147,148]. Immunodeficient mice also support the long-term survival of transplants in the eye [149,150]. The eye exhibits a close interface with meningeal lymphatics of the optic nerve and is subject to retinal immune surveillance. However, the neuroretina possesses mechanisms to avoid tissue damage through immune reactions by suppressing or neutralizing peripherally derived immune mediators [151,152]. Strikingly, migrating ocular APCs are capable of inducing immune tolerance not only for retina-derived antigens, but also for antigens draining from grafts of the anterior orbit or subretinal zone; this result is obtained by inducing T regulatory cells in the spleen that suppress the adaptive immune response [151]. This mechanism functions to avoid peripheral recognition of any draining “ocular” antigens, thus protecting ocular physiology, and incidentally confirming the drainage of ocular antigens to the periphery.

Hence, recent and ongoing studies extend and renew the classic definition of CNS immune privilege by introducing the concept of dynamic and close communication between the CNS and periphery, driven by fluid efflux along the glymphatic and meningeal lymphatic vessels, to present antigens at distal sites.

### 3.1. The Glymphatic and Meningeal Lymphatic System as Key Players during Neuroinflammation

Drainage of fluid and antigens from the CNS via the glymphatic system and the meningeal lymphatic vessels prompts a redefinition of the CNS as an immunologically privileged site [15,16,141,142,153,154,155]. Traditionally, CSF was believed to be absorbed by arachnoid granulations into the venous blood, but it is now evident that CSF outflow in rodents mainly occurs through the meningeal lymphatic system [15,16,156]. Those studies demonstrated the existence of lymphatic vessels in the dura, along with arteries and venous sinuses in mice [15,16]. Interestingly, the meningeal lymphatic vessels drain the CSF and ISF downstream of the glymphatic system (Figure 1 and Figure 2) [18,157].

The meningeal lymphatic vessels carry peripheral immune cells, cytokines, and CNS-derived antigens out of the cranium via, for example, peri-sinus locations to the foramina at the base of the skull [18,155,158]. The final destination for drainage of antigens from the brain parenchyma is the deep cervical lymph nodes [16,17,158,159,160]. Exogenous antigens such as keyhole limpet hemocyanin, upon injection into the CSF, are transported via the CSF into the subarachnoid space and drain in the deep cervical lymph nodes some hours later [143]. Around four days after antigen administration, antigen-specific antibody-secreting cells are found in the superficial and deep cervical LNs [143]. Notably, before reaching the deep cervical lymph nodes, some of the ISF/CSF, amongst other paths, drains through the cribriform plate and into the nasal mucosa [10,160,161].

The glymphatic and meningeal systems thereby act as a drainage route for CSF and simultaneously contribute to the immune surveillance of the CNS. Considering recent findings for an immune-regulating role of the meningeal lymphatics, the emergence of CSF spaces for immune surveillance of the CNS and their role in peripheral CNS antigen recognition presents a novel perspective [18]. That latter study demonstrated the dural sinuses to be a neuroimmune interface where antigens from the CNS accumulate and activate antigen-presenting cells (APCs) to further initiate adaptive immune responses in mice. T cells and APCs were elsewhere shown to exist in the dural sinus areas in humans, implying that this might serve as an attractive site for potential blockage of immune cell entry as a therapeutic strategy in MS, for example [63]. Strikingly, ligation of cervical lymph vessels reduced drainage of CSF in a mouse model of glioblastoma [162]. This ligation abolished access of peripheral immune cells to the tumor site, resulting in a more aggressive growth of the tumor due to the lack of peripheral immune cells [162]. Interestingly, another study showed that photoablation of dorsal meningeal vessels in young mice resulted in learning and memory deficits, which was apparently due to impaired CSF perfusion [159], highlighting the importance of CNS drainage for cognitive function. The glymphatic system links functionally to the lymphatic system connecting deep brain tissues to the immunological active periphery. This pathway challenges the concept of immune privilege of the brain parenchyma. CSF bulk flow carries cytokines and other immune signals from the parenchyma, as demonstrated in earlier studies following the efflux of antigens placed in the parenchyma [143,144], and also in a model of meningeal lymphatic drainage of CSF tracer drainage [163].

### 3.2. CNS Innate Immune Response during Neuroinflammation

With the discovery of the glymphatic and meningeal lymphatic system, it is now clear that antigens are drained from the CNS in a manner remarkably similar to antigen clearance in the lymphatic system. However, the lymphatic system in the periphery drains solutes and waste nonselectively, such that all antigens can reach the lymph nodes. On the other hand, CNS resident and perivascular immune cells are positioned to phagocytose and degrade proteins excreted by or drained from the brain parenchyma. Thus, there is limited immune trafficking and surveillance, which grants the CNS parenchyma a specialized and partially privileged immune status. At least 400–600 mL of CSF leaves the brain daily [164,165,166], but only traces of CNS-derived immunogenic substances are drained to the periphery, and this without provoking any peripheral immune reaction under physiological conditions [154]. The tight physiological barriers of the CNS and the effective intrinsic clearance via the meningeal lymphatics [18] together exert a strict selective control of the antigens that ultimately exit the CNS and reach the periphery. In addition, we attribute importance to the cervical lymph nodes in supporting an immunological tolerance towards CNS and nasal mucosa-derived antigens [167]. Furthermore, we suspect that the meninges also possess intrinsic tolerogenic mechanisms to avoid autoimmunity.

Compared to other body fluids (e.g., synovial, peritoneal, pericardial, and pleural fluids), the CSF has a lower abundance of immune cells. Crossing of the BBB by immune cells in the absence of neuroinflammation is restricted to a few activated T cells in rodents [18,142]. Indeed, a higher fraction of adaptive immune cells is present in the CSF in comparison to innate immune cells in healthy individuals [168]. The predominant cells in the CSF are central memory CD4+ T cells (Tcm), pointing towards a role of CNS surveillance via the CSF, rather than an active role of immune cells with effector functions [168]. It was hypothesized that Tcm monitors the CNS within the subarachnoidal and meningeal spaces, encountering perivascular macrophages at the brain interfaces [169]. However, it was recently shown that APCs sample CNS-derived antigens to T cells and myeloid cells in peri-sinus locations [18]. More specifically, a systemic interconnection of the CNS and meningeal lymphatics has been described in which antigens injected into the cisterna magna flow through the glymphatic system and are presented to the APCs in peri-sinus areas, where they may possibly activate adaptive T-cell immune response [18]. Hence, such observations call into question the classic concept of immune privilege of the CNS parenchyma; certainly, the CNS is immunologically more active than first envisioned by Medawar. The discovery of the meningeal lymphatic vessels revealed how immune cells can traffic within the CSF and establish a direct contact between the meningeal lymphatics and the periphery [157]. It is currently believed that T cells enter the meninges and CSF through the choroid plexus, blood vessels in the leptomeninges, or meningeal blood vessels in the dura [168,170]. CSF is therefore a transportation route, similar to blood vessels, allowing T cells to rapidly reach damaged or inflamed areas of the CNS, while also acting as a storage space or buffer that prevents potentially damaging effector T cells from entering the brain parenchyma, since integrin adhesive forces are only triggered if activated T cells meet their antigen [170]. We suspect that intrinsic tolerogenic mechanisms must exist in the meninges, as immune activation would otherwise occur and induce autoimmunity [171]. Further research into revealing the factors that dampen immune reactions or stops them entirely is highly important for obtaining a better understanding of the immune interface, in terms of immune privilege and in relation to future therapeutic targets.

**Figure 3:**
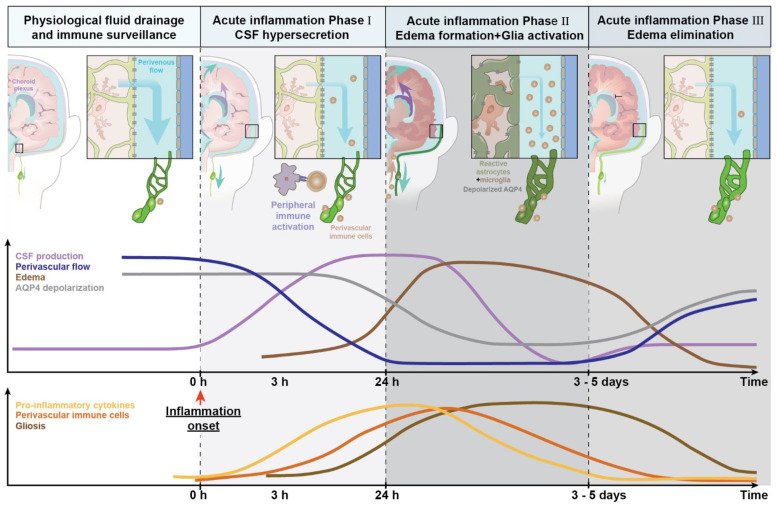
The effect of acute inflammation on the glymphatic and meningeal lymphatic system.

As described in Section 2.1, CSF production and glymphatic clearance are affected during systemic inflammation. We speculate that, depending on the severity of the inflammatory insult, there is rapid onset of a transient CSF hypersecretion (Figure 3). This CSF hypersecretion may support a rapid export of secreted cytokines/chemokines by activated glial cells (phase I, Figure 3). Astrocytes will simultaneously increase their AQP4 expression, but the additional AQP4 channels are not polarized towards the vascular endfeet and therefore do not support unidirectional glymphatic transport. CNS-derived cytokines/chemokines and antigens can then reach the peripheral lymph nodes, triggering immune recruitment. Subsequently, accumulation of waste, immune mediators, and stagnating fluid drainage due to the swelling of meningeal lymphatic vessels all contribute to tissue swelling or edema (phase II, Figure 3), which is ultimately followed by edema resolution and recovery to normal state (phase III, edema elimination). Most studies suggest that the reactive changes in glial cells partly persist (phase II, Figure 3), and thus chronically impair brain fluid transport.

The top panel shows changes in CSF production over the time course of inflammation. The insets on the left of the coronal view of the human brain depict the macroscopic changes in fluid dynamics of perivenous spaces and meningeal lymphatic vessels (drainage pathway). The time graph below depicts changes in CSF production, perivenous fluid flow, edema formation, and AQP4 mislocalization over time. The schema in the bottom part of the figure illustrates the change of pro-inflammatory cytokine secretion, accumulation of immune cells, and gliosis over the time course of acute inflammation. The steady-state of **fluid drainage and immune surveillance during physiological and homeostatic conditions** is presented on the left side (white). Lymphatic vessels and lymph nodes (green), choroid plexus (purple), and brain fluid transport (blue arrows indicates the magnitude of periarterial CSF inflow). **During phase I, acute inflammation** (light grey), the upregulation of MHC molecules, and an increased production of inflammatory cytokines induces hypersecretion of CSF and activation of glial cells, resulting in an increased shunting of CSF out of CNS via the meningeal and cervical lymphatic vessels. **Phase II of acute inflammation, edema formation** (dark grey) sharply reduces glymphatic transport due to a combination of factors, including immune cell accumulation in the perivascular spaces and edema. The choroid plexus hypersecretion of CSF results is an increase of shunting of CSF out of CNS. The inserts and the time schema show gliosis with loss of AQP4 polarized expression in the vascular endfeet of astrocytes and a reduction in perivascular CSF influx. **Phase III, edema elimination** (light grey), is linked to decreased CSF secretion in conjunction with a more polarized expression of AQP4 in the endfeet, supporting edema resolution and a gradual return towards normal conditions, albeit seldom with complete return of homeostasis.

## 4. Conclusions

The pivotal role of the astrocytic water channel AQP4 for the function of the glymphatic system demonstrates the crucial role of astrocytes, not only on a local scale by interaction with other CNS resident cells, but in in a broader role by maintenance of CNS fluid homeostasis. With the discovery of the glymphatic [2,3,4,5] and meningeal lymphatic systems [15,16] and the recently described neuroimmune interface [18], we have revisited the classic definition of immune privilege of the CNS. The original theory of immune privilege has been questioned or revised with the discovery of antigen drainage from the parenchyma via the glymphatic and the meningeal lymphatic system [18]. Originally described as lacking immune response to (and rejection of) transplanted tissue grafts, it is now known that brain grafts are indeed rejected and able to locally trigger immune response [145]. Experimental transplantation models require the complete absence of a functional adaptive immune response to ensure long term graft survival [146,147,148]. For ocular transplants, it is known that peripheral circulating eye-derived APCs induce peripheral immune tolerance [151], and similar mechanisms may well exist for the brain interface. Yet the exact mechanisms of the meningeal lymphatic interface [18] and the glymphatic system [3] during/in CNS immune surveillance are at present only scantly understood. The neuroimmune interface located in the meninges might actively select antigens draining from the brain parenchyma facilitated by the glymphatic system [18]. Furthermore, APCs in the meningeal lymphatic vessels likely play a crucial role in CNS immune surveillance. These findings emphasize the existence of dynamic and complex communication between CNS structures and the periphery. We suspect that tolerogenic mechanisms exist in the meningeal neuroimmune interface (for both brain and eye), which ensure CNS immune surveillance and protect against peripheral autoimmune responses against CNS structures; MS may prove to be an instance of failure of these mechanisms. By today, we can conclude that the brain and the eye indeed exhibit local specialized immunological mechanisms, which are under tight control and shielded from peripheral interactions through barriers (BBB and BRB) that impart relative but not absolute immunological privilege.

## 5. Future Perspectives

There is little doubt that glymphatic fluid transport is suppressed in acute and chronic neuroinflammatory conditions [27,37,70,115]. Depending on the nature of the inflammatory insult, stagnating fluid drainage will then contribute to edema formation, cytokine accumulation, and reactive responses of microglia, astrocytes, and/or neurons. Astrocytic AQP4 water channels are upregulated during this process, but their mislocation away from the vascular endfeet during inflammatory suppresses, rather than facilitates, polarized glymphatic flow [27,60,172,173,174,175].

Neuroinflammatory and neurodegenerative diseases impose an enormous and ever-growing economic burden, now causing millions of deaths every year worldwide [176,177,178]. It is clear that the glymphatic system is affected in chronic neuroinflammatory diseases such as Alzheimer’s disease [27,42], and could therefore be a potential therapeutic target. One approach for targeting the glymphatic system and the downstream meningeal lymphatic system could be to administer drugs into the CSF to dampen the reactive responses of microglia and astrocytes. The BBB can be bypassed by using the intrathecal route for direct delivery of therapeutics into the CSF via cisternal spaces [179]. Indeed, the glymphatic system has previously been shown to support drug delivery to the CNS, even extending to antibodies targeting amyloid-β [180]. In this regard it is important to recall that certain anesthesia regimens and hyperosmolar therapy can enhance glymphatic drug delivery, resulting in, e.g., increased delivery of an amyloid-β antibody to the brain parenchyma [180,181].

Conversely, slowing brain drainage might also be used therapeutically in certain circumstances. Impeding solute clearance could potentially slow the removal of therapeutic agents like anti-cancer drugs, immunotherapy, and immune modulators. In this regard, one should be mindful that glymphatic clearance peaks at night, such that the circadian clock should be taken into consideration when administering intrathecally delivered medications [42]. It could be of interest to strengthen existing pathways for the entry of immune cells into the CNS to fight off tumors, as seen in a mouse model of glioblastoma [162].

We do not yet have an adequate understanding of the mechanisms whereby CSF hypersecretion affects the glymphatic system. However, in other conditions, glymphatic flow has been shown to function independently of CSF production [182], suggesting that the excess CSF may be directly shunted out via lymphatic vessels in the context of acute inflammation. Targeting of Toll-like receptor 4 and its downstream mediators has been suggested as a therapeutic strategy for inflammation occurring in posthemorrhagic brain injury [183]. Whether a similar approach would be beneficial in other neuroinflammatory diseases manifesting with brain edema would be an interesting topic for research. Multiple lines of work have clearly documented that the functioning of the glymphatic and meningeal lymphatic systems is affected by neuroinflammation, which may be exacerbated via a feedforward mechanism. Obtaining a more complete understanding of how neuroinflammation interacts with brain fluid transport may aid in developing new therapeutic targets to combat acute inflammatory events, with implications for the pathogenesis of neurodegenerative diseases.

## Abbreviations

AD	Alzheimer´s disease
APC	Antigen-presenting cells
AQP4	Aquaporin-4
BBB	Blood–brain barrier
BRB	Blood–retina barrier
CM	Cisterna magna
CNS	Central nervous system
CSF	Cerebrospinal fluid
IOP	Intraocular pressure
ISF	Interstitial fluid
LPS	Lipopolysaccharides
MRI	Magnetic resonance imaging
MS	Multiple sclerosis
NMO	Neuromyelitis optica
NMOSD	Neuromyelitis optica spectrum disorders
NKCC1	Na+, K+, Cl− cotransporter
TBI	Traumatic brain injury
Tcm	Central memory T cells
TLR-4	Toll-like receptor 4
